# A Rare Case of Phlegmonous Gastritis in a Previously Healthy Male: A Case Report

**DOI:** 10.7759/cureus.35013

**Published:** 2023-02-15

**Authors:** Omar M Masarweh, Raj Shah, Feras Al-Moussally, Andy Huang, Basher Atiquzzaman

**Affiliations:** 1 Department of Internal Medicine, University of Central Florida, Hospital Corporation of America (HCA) Healthcare, Orlando, USA; 2 Medicine, University of Central Florida College of Medicine, Orlando, USA; 3 Internal Medicine, University of Central Florida, Orlando, USA

**Keywords:** diffuse phlegmonous gastritis, purulent emesis, gastric wall thickening, antibiotics therapy, microabscess, upper endoscopy, gastritis, phlegmonous gastritis

## Abstract

Though rare, phlegmonous gastritis (PG) is a serious and life-threatening infection of the gastric submucosa and mucosa. Many factors have been associated with PG, including malignancy, chronic alcohol use, and immunocompromised states. Clinical presentation of PG is often non-specific, and diagnosis is often delayed. Early recognition and starting antibiotics significantly reduce overall mortality. We describe a case of a previously healthy male who presented with moderate abdominal pain and was found to have PG that was treated with an extensive course of antibiotics and total parental nutrition. Contrary to previously described cases in the literature, our patient had no predisposing factors, highlighting the importance of suspecting PG even in the absence of such factors and demonstrating the effectiveness of antibiotics in this disease.

## Introduction

Phelgmonous gastritis (PG) is a disease with subtle and nonspecific clinical manifestations resulting in a delay in diagnosis and treatment, which increases mortality. Symptoms such as abdominal discomfort, nausea, vomiting, anorexia, and fevers are some of the most common symptoms reported with PG, along with predisposing factors such as alcohol use, previous history of gastritis, bacteremia, malignancy, or recent endoscopic manipulation. Although rarely seen, purulent emesis is considered to be pathognomonic. It is thought to be due to bacterial invasion into the gastric submucosa either from direct invasion or via bacteremia [1,2]. Due to its rarity, we intend to present a case of PG in a previously healthy male who responded well to antibiotics. This case highlights the need to have PG as a possible differential in patients presenting with nonspecific symptoms of abdominal discomfort even without the presence of typical risk factors, such as our patient.

This article's abstract was presented at the American College of Gastroenterology Conference in October 23, 2022.

## Case presentation

**Figure 1:**
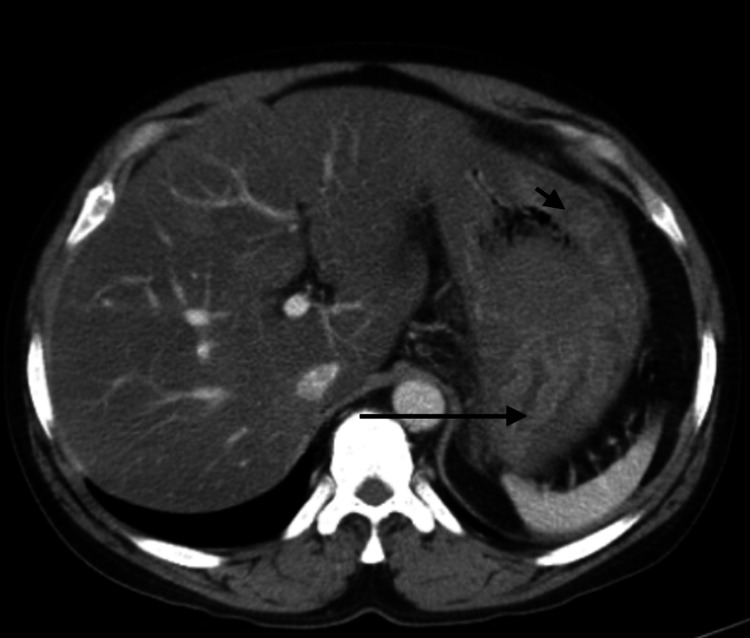
Computed tomography of the abdomen with contrast showing increased gastric wall thickness (arrowhead) and prominent gastric folds (arrow)

**Figure 2:**
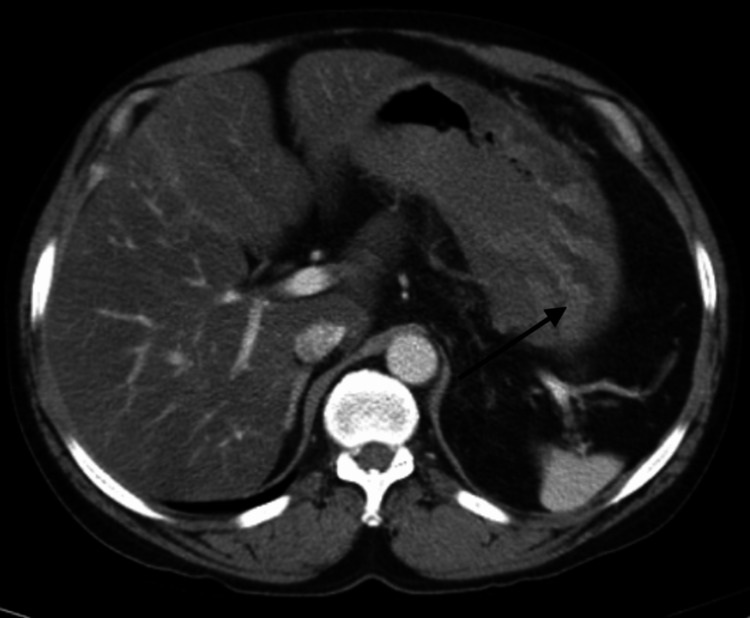
Computed tomography of the abdomen with contrast showing increased gastric wall thickness and prominent gastric folds (arrow)

**Figure 3:**
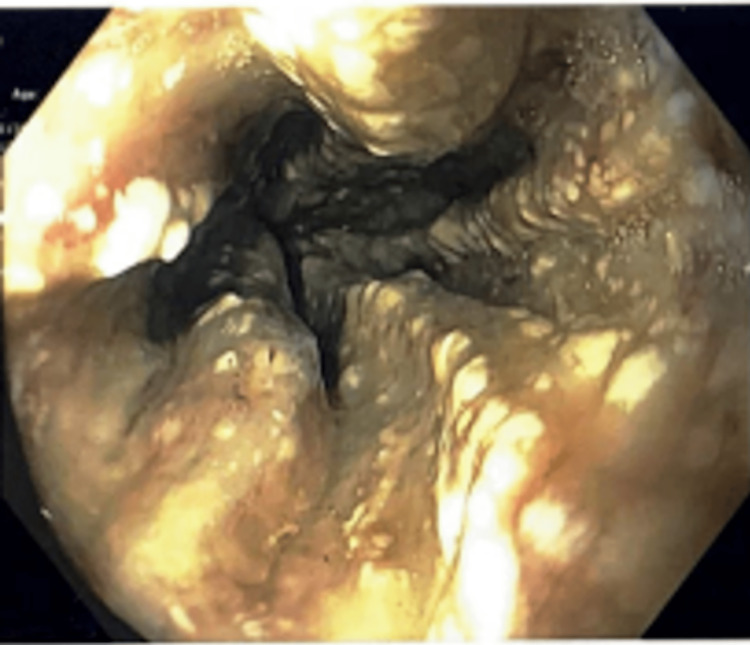
Upper esophagus showing severe esophagitis

**Figure 4:**
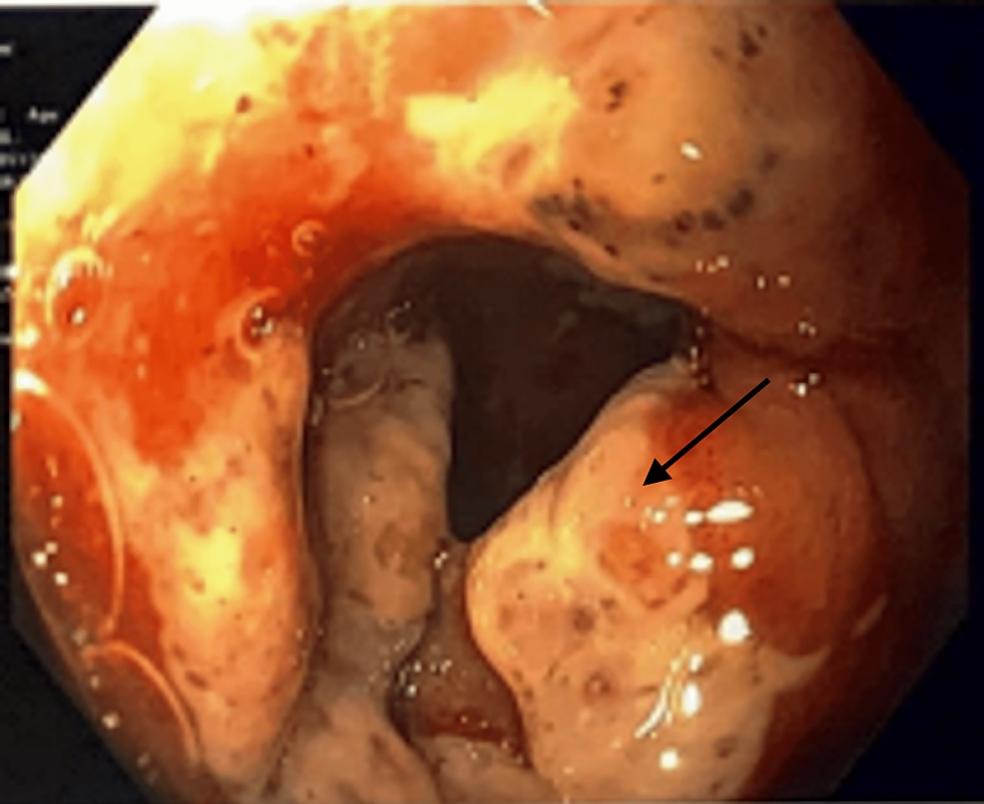
Esophagogastroduodenoscopy showing severe ulcerative duodenitis in the proximal duodenum (arrow)

**Figure 5:**
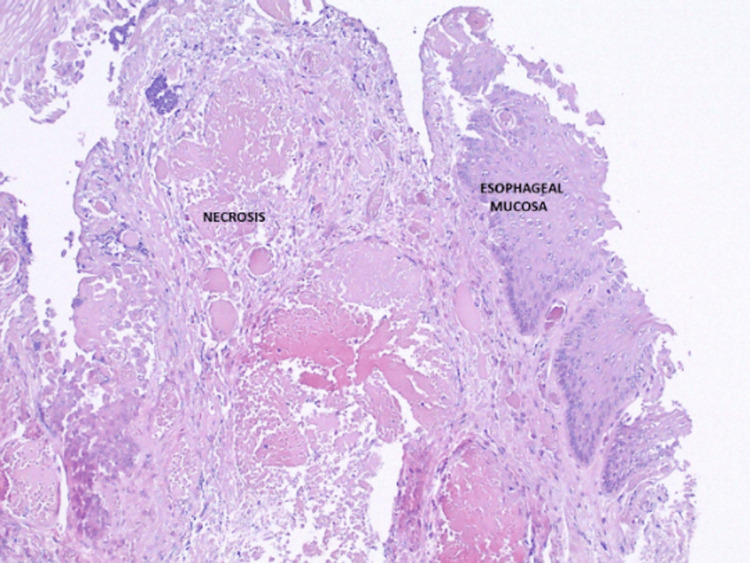
Esophageal biopsy showing an abrupt transition from normal tissue to necrosis that reflected areas of necrosis seen on EGD EGD - esophagogastroduodenoscopy

**Figure 6:**
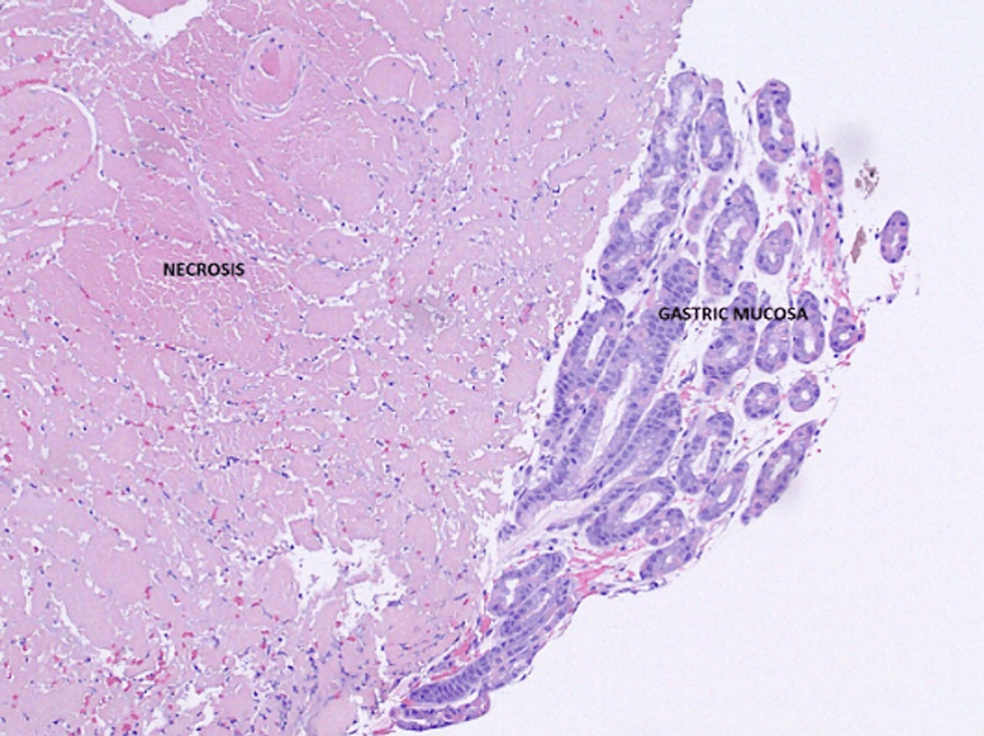
Areas of patchy necrosis in the gastric biopsy showing an abrupt transition from normal tissue to necrosis

**Figure 7:**
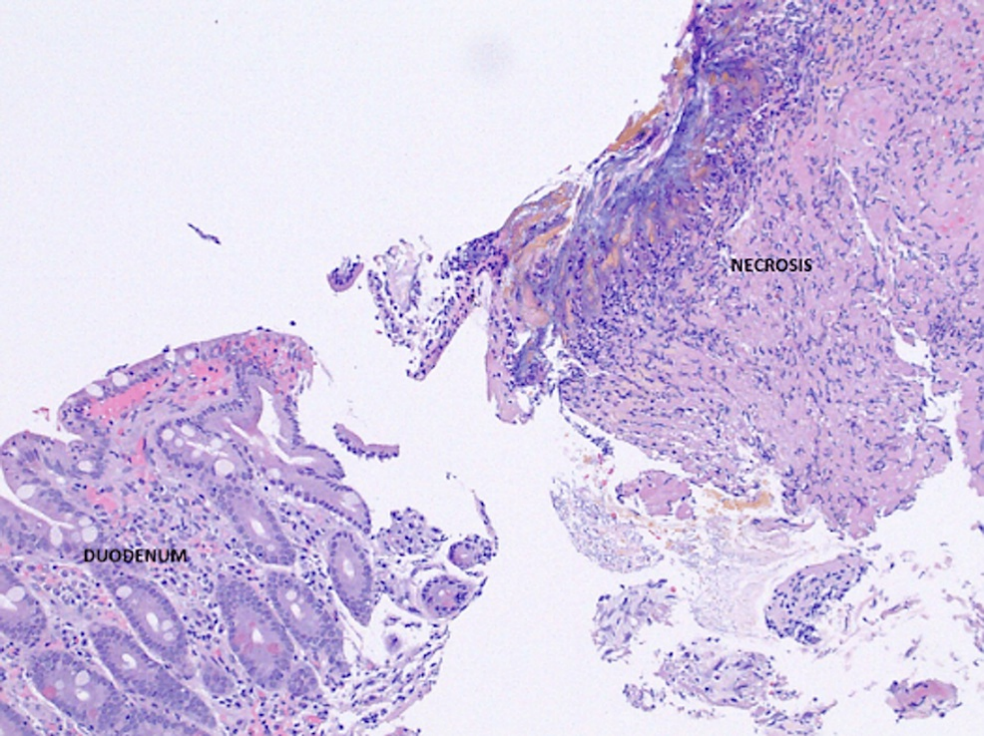
Biopsy of the duodenum showing the abrupt transition from normal tissue to necrosis that was seen on EGD EGD - esophagogastroduodenoscopy

A 55-year-old male with a history of hypertension and hyperlipidemia presented to an emergency department with complaints of odynophagia, intractable nausea, and coffee-ground emesis for one day. He denied a history of alcohol use, smoking, illicit drug use, or nonsteroidal anti-inflammatory drug use. On arrival, blood pressure was 132/91 mmHg, heart rate was 86 beats per minute, temperature was 97° F, saturating 97% on room air. Physical exam was remarkable for diffuse abdominal tenderness but without guarding or signs of acute abdomen. Laboratory investigation showed a white cell count of 19,500/mcL (normal 4,000-11,000 cells/mcL) with a neutrophil predominance of 70.8% (reference range 34-67.9%), hemoglobin 17g/dL (normal 13-16g/dL), erythrocyte sedimentation rate46mm/hr (normal 0-15mm/hr) and was negative for HIV, syphilis, and hepatitis A, B, and C. Blood cultures were obtained and remained negative. Contrast-enhanced computed tomography (CT) of his abdomen with contrast showed thickening of the gastric wall with prominent gastric folds, however no signs of any biliary etiology such as gallbladder wall thickening or pericholecystic fluid (Figures 1-2). He was empirically started on vancomycin, piperacillin/tazobactam, and pantoprazole due to his leukocytosis and presumed abdominal infection. Esophagogastroduodenoscopy (EGD) showed severe diffuse esophagitis, although worse in the lower esophagus, gastritis, and ulcerative duodenitis with prominent gastric folds and excessive purulent debris with areas of micro-abscesses (Figures 3-4). Biopsies obtained during EGD showed necrosis in the esophagus, stomach, and duodenum with areas of fibrin thrombi and eosinophils (Figures 5-7). Immunochemical and special stains were negative for *Helicobacter pylori (H. pylori)*, negative for malignancy with CD 68, Cytomegalovirus, negative for amyloid on congo red, and fungi with Periodic Acid-Schiff stains. Vasculitis was suspected due to the areas of patchy necrosis, and he was given a short course of prednisone. It was shortly discontinued due to a negative autoimmune workup consisting of a negative anti-cyclic citrullinated peptide, antinuclear antibody, cytoplasmic antineutrophil cytoplasmic autoantibody, perinuclear antineutrophil cytoplasmic antibodies, topoisomerase-1 antibody, anti-centromere antibody, anti-RNA polymerase III antibody, and complements three and four, with rheumatoid factor elevated at 15 IU/mL (normal 0-12 IU/mL). During the admission, he received broad-spectrum antibiotics with vancomycin and piperacillin/tazobactam and received total parenteral nutrition for 36 days until he could tolerate oral intake. Multiple attempts were made to transition to an oral diet; however, the patient continued to have nausea and vomiting. A gastric emptying study and CT of the abdomen and pelvis with oral contrast were done to help establish a possible cause, but both were negative for gastric outlet obstruction or small bowel obstruction. Fortunately, he was eventually discharged home, tolerating a soft diet; however, the patient did not follow up outpatient for a follow-up EGD.

## Discussion

Acute PG is a very rare disease that has been limited to mostly one case report a year over the last century. Due to its rarity and its non-specific clinical presentation, PG might be missed when evaluating a patient with abdominal pain. Although it can affect any person of any age, it is more commonly seen among adults in their 50-70s, with a 2:1 ratio affecting males more often than females [3]. PG's previous mortality rate was as high as 92%, but with the emergence of antibiotics, it has decreased the mortality to under 40%. Early detection and swift initiation of antibiotics has led to such a drastic decline [4].

The exact cause and mechanism of PG are not fully understood; however, a few theories have gained traction over the last few years. At present, it is thought that bacteria invade the gastric lining either through a compromised barrier such as a peptic ulcer, damage from endoscopic manipulation or even after feeding tube placement that leads to direct invasion of the gastric mucosa and submucosa by bacteria [3]. Another mechanism is a bacterial invasion in a host with a pre-existing condition such as chronic alcohol use, immunocompromised states such as HIV, and malignancy. Additionally, lymphatic spread from intra-abdominal infections, such as cholelithiasis, appendicitis, or through bacteremia, have also been proposed mechanisms of spread. Although hemolytic streptococcus accounts for 70% of cases, other organisms such as *Staphylococcus aureus*, *Pneumococcus*, *Enterococcus*, and polymicrobial infection have also been isolated [4]. Interestingly, in our case, there was no isolated organism either through blood culture or from specimens obtained via EGD.

PG is categorized into two subgroups based on lesion range, localized and diffuse. Diffuse PG has a higher overall mortality compared to localized PG (54% vs. 10%, respectively) [3-5]. Diffuse PG is more common and characterized by a generalized thick, dark, and red gastric wall with purulent discharge when pressure is applied to the gastric wall. On the other hand, localized PG is characterized by gastric mucosal ulceration, hyperemia, erosion, necrosis, and possibly bleeding. In our patient, the gastric wall was obviously thickened, and EGD demonstrated purulent discharge in the esophagus, stomach, and duodenum, therefore, classifying it as a diffuse type.

Purulent emesis is pathognomonic, however rarely seen [1,3]. In order to diagnose PG, high clinical suspicion and radiographic evidence can help tailor the clinical course and further management and diagnostic evaluation. Imaging such as a CT scan is a great initial test that can detect gastric wall thickening; however, it is not routinely recommended, and most patients warrant direct visualization and biopsy for accurate diagnosis. Endoscopic findings include fibrinopurulent exudates that line the stomach, erosions of the stomach that can extend to the duodenum, and edematous mucosa [5]. Histopathologic findings can include neutrophils, plasma cells, and intramural hemorrhage with necrosis [1,6-8].

The cornerstone of the treatment of PG is antibiotics. As awareness grew, surgical interventions such as gastric resection also became an option for severe diffuse disease. It is difficult to assess if antibiotics or surgical resection is superior; however, multiple reviews have suggested that it is reasonable to start with antibiotics and reserve surgical resection for patients who develop complications or who fail to improve [3,9,10]. Therefore, broad-spectrum antibiotics should be initiated on admission since resistant bacteria and polymicrobial infections are common, and with a plan to tailor antibiotics if an organism is isolated. Unfortunately, there are no guidelines to direct antibiotic length or further management beyond clinical improvement. More studies and reports are needed in order to establish appropriate guidelines to identify and manage this disease. Although there is no consensus as to proper follow time and procedure, it is wise to have follow-up EGD to ensure healing, clearance of disease, as well as possibly identifying a malignancy or ulcer that may have been difficult to detect during active disease.

## Conclusions

Due to PG's relative rarity and non-specific clinical presentation, early diagnosis often is a challenge which is one reason why it is often fatal. PG is due to bacterial invasion into the gastric wall, including the submucosa, and possible further invasion into the mucosa and serous membrane. Such invasion could be a result of lymphatic spread from intra-abdominal infections, or a compromised mucosal wall as is the case in gastritis. This case is one of the few reported cases in the literature where PG presents in a previously healthy individual without comorbidities. Initial treatment is usually with antibiotics, with surgical intervention being an option if medical management fails. Further studies and research are needed in order to establish clear diagnostic criteria and management guidelines for acute care as well as long-term surveillance. 
